# Access to Difficult-to-reach Population Subgroups: A Family Midwife Based Home Visiting Service for Implementing Nutrition-related Preventive Activities – A Mixed Methods Explorative Study

**DOI:** 10.3934/publichealth.2015.3.516

**Published:** 2015-08-21

**Authors:** Helena Walz, Barbara Bohn, Jessica Sander, Claudia Eberle, Monika Alisch, Bernhard Oswald, Anja Kroke

**Affiliations:** 1 Fulda University of Applied Sciences, Department of Nutritional, Food and Consumer Sciences, Fulda, Germany; 2 formerly: Fulda University of Applied Sciences, Department of Nutritional, Food and Consumer Sciences, Fulda, Germany; currently: University of Ulm, Institute of Epidemiology and Medical Biometry, ZIBMT, Ulm, Germany; 3 Fulda University of Applied Sciences, Department of Nursing and Health Sciences, Fulda, Germany; 4 Fulda University of Applied Sciences, Department of Social Work, Fulda, Germany; 5 Youth Welfare Office, Quality Management / Early Prevention, Fulda, Germany

**Keywords:** child health services, family research, socioeconomic factors, midwifery, public health, perinatal programming, health inequality, home visiting services

## Abstract

Health and social inequality are tightly linked and still pose an important public health problem. However, vulnerable and disadvantaged populations are difficult to reach for health-related interventions. Given the long-lasting effects of an adverse, particular nutrition-related, intrauterine and neonatal environment on health development (perinatal programming), an early and easy access is essential for sustainable interventions. The goal of this explorative study was therefore to elucidate whether an existing access of family midwives (FMs) to families in need of support could be an option to implement effective public health and nutrition interventions. To that end three research objectives were formulated: (1) to determine whether a discernible impact of home visits by FMs can be described; (2) to identify subgroups among these families in need of more specific interventions; (3) to determine how relevant nutrition-related topics are for both FMs and the supported families. For addressing these objectives a mixed methods design was used: Routine documentation data from 295 families visited by a family midwife (FM) were analyzed (secondary analysis), and structured expert interviews with FMs were conducted and analyzed. Study reporting followed the STROBE (STrengthening the Reporting of OBservational studies in Epidemiology) statement. Based on the FMs reports, a significant improvement (p < 0.001) regarding psycho-social variables could be determined after the home visits. Single mothers, however, seemed to benefit less from the FMs service compared to their counterparts (p = 0.015). Nutritional counseling was demanded by 89% of the families during the home visits. In addition, nutrition-related topics were reported in the interviews to be of high interest to both families and the FMs. Based on the obtained results it is concluded that FMs home visits offer a promising access to vulnerable and disadvantaged families for implementing nutrition-related preventive activities.

## 1. Introduction

Numerous studies and reviews describe the tight link between social status and health [1]–[8]: a low socio-economic status (SES) has been shown to be positively associated with health-risk behaviors such as smoking [9]–[12], low physical activity [13]–[15] or unhealthy diet [16]–[18]. Moreover, chronic diseases such as cardiovascular disorders [19], [20], type 2 diabetes mellitus [1], [6], [21] and certain types of cancer [22] occur more frequently in these groups, resulting in a lower life expectancy compared to people with a higher SES [23]. Furthermore, psycho-social problems increase with decreasing social status. Most importantly, these inequalities in health are already evident in childhood and have considerable health and (psycho-) social consequences [24]–[26]. Therefore, children from vulnerable and disadvantaged families are more frequently affected by developmental disorders, dental problems as well as mental and behavioral disorders [24]–[28]. They also perceive their own health and health-related quality of life as reduced, and they more frequently develop health-risk behaviors such as smoking, physical inactivity or inappropriate diet later in life [24]–[28].

Explanations for this tight link between social status and health encompass economical, psycho-social, behavioral as well as biological aspects [29]–[31]. On the biological level, "perinatal programming" plays an important role. According to this concept, early life influences determine important metabolic and functional processes irrespective of the individual genetic disposition [32]. The time window from pregnancy to approximately the end of the 2nd year of life represents a particularly critical stage of development. During this phase the growing organism is characterized by developmental plasticity allowing external influences to exert life-long effects [33]. In this context, numerous epidemiological, clinical and experimental studies have documented the quality and quantity of pregnant women's nutritional status [34], [35], dietary intakes during pregnancy [36]–[39] and infant nutrition [40]–[43] as fundamental factors leading to life-long imprinting. Thereby, perinatal programming might contribute to the transgenerational fixation of health and social inequality within families.

Given the problems outlined above, the reduction of health and social inequality is of major public health interest [44], [45]. Accordingly, the World Health Organization emphasizes that a major focus should be put on early child development to diminish the social gradient regarding health outcomes [46]. While intervention studies that focus on gestational weight gain [47], [48], cessation of maternal smoking [49], [50], dietary intake during pregnancy [51], maternal stress reduction [52] or on breastfeeding promotion [53], [54] indicate the potential to positively influence health related behavior of pregnant women and related outcomes in the offspring, the participation of vulnerable and disadvantaged population groups in such studies is very limited [55]. An important cause is the difficult reachability and low motivation to participate [56]–[59]. Hence, an access to vulnerable and disadvantaged families, especially during pregnancy and early parenthood, is imperative for targeted and effective early interventions. This access is characterized by targeting groups in their setting, focusing on their needs and allowing a fast as well as non-bureaucratic service access. Using an already existing access via social assistance programs could be an option to implement additional health-and nutrition-related interventions for families in need of support [60]. Similar to internationally known “home visiting services” [61]–[68], activities of the “Early Prevention” (EP) projects in Germany offer intensive pre- and postnatal care via family midwives (FMs) for vulnerable and disadvantaged families by visiting them in their home [69]–[71]. These services offer support to families (especially to high risk groups) by trained staff at their home [65].

However, the proposed option to further use this existing access for implementing extended health-and nutrition-related interventions has not been investigated so far. Therefore, the goal of this study was to elucidate whether the existing access of FMs to these difficult to reach population subgroup is an option for additional health and nutrition intervention delivery. Research objectives of this study were (1) to determine whether a discernible impact of home visits by FMs can be described; (2) to identify subgroups among these families in need of more specific interventions; (3) to determine how relevant nutrition-related topics are for both FMs and the supported families.

## 2. Materials and Method

**Figure 1.**
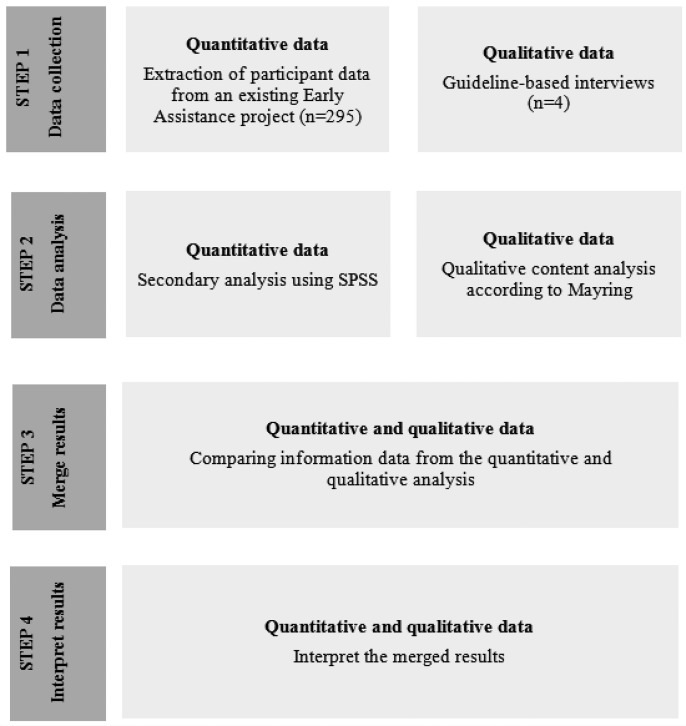
Flowchart of the convergent mixed methods design (according to Creswell et al. 2010 [74])

Reporting of this study is presented according to the STROBE (Strengthening the Reporting of Observational Studies in Epidemiology) statement [72], [73]. The so-called convergent mixed methods approach according to Creswell [74] was used to address the various objectives of these analyses. Accordingly, qualitative and quantitative data were obtained separately and combined afterwards [74]. **Figure 1** visualizes the combining procedure. All staff involved in data collection and/or data analyses were obligated to the German data protection rules and signed a respective obligation form.

### 2.1. Project setting

EP projects were initiated in Germany in 2006 to particularly prevent child neglect and maltreatment [75]; the “Law on Child Protection” provides their legal foundation. The EP project in the study region Hesse, forming the basis for the current analysis, has been introduced in 2008 after a regional network conference. It focuses on families with identified need of support, e.g. due to financial or psycho-social problems. The primary main objective of these EP project is to “*constitute or ensure sufficient skills in the fields of education, child care and support for mothers and fathers in order to provide adequate development conditions for their children*”. The project region has a population of around 217,000 with annually about 1,800 births.

FMs home visits are the core activity of the EP projects. These visits offer an extended midwife care (free-of-charge) compared to the regular (free-of-charge) midwife support. Visits are scheduled up to 1 year postnatal, as compared to 8 weeks in the regular service.

Midwives with at least 5 year professional experience are further qualified to become FMs. During a 1 year period 14 modules, covering topics such as public health, legislation, pedagogy, psychology, and pediatrics have to be attended. After successful completion of the training FMs are required to regularly attend advanced training courses as well as supervisions [76]. The focus of FMs counseling currently lies on psycho-social support via home visits during pregnancy and/or the first year of a child's life. FMs service can either be requested by the family in need or suggested by medical or social institution staff (e.g. gynecologist, hospital, maternity wards, social counseling staff). Given the families consent, 20 home visits during a one-year period (also free-of-charge) are offered, starting either pre- or postnatal. In case of continued need of support, additional 20 visits are possible. FMs are self-employed midwives who are paid per case by the Youth Office. A FM is looking after 0–9 families simultaneously, depending on her available time and the number of families in need. Average duration of a visit is 60 minutes.

### 2.2. Quantitative data

Quantitative data were obtained from existing data sets collected from routine project documentation activities of the EP project. Anonymous data were provided by the local youth welfare office. The data analyses followed the German guidelines and recommendations for "Good Practice Secondary Data Analysis” [77]. As a secondary analysis a pre- and post-intervention comparison was performed. The primary aim of data collection was to document progress in consideration to psycho-social variables during the FMs support.

From a total of 336 EA participants, complete anonymous data from 295 (87.8%) participating families, collected between April 2009 and May 2013, were available. Due to incompleteness of data the remaining 41 (12.2%) participants had to be excluded from this analysis. A comparison of included with excluded subjects showed no significant differences regarding socio-demographic variables (e.g. maternal year of birth, country of origin, partnership status). Data collection was performed by the FMs after obtaining informed consent of the families. Following standardized procedures, basic information and general characteristics of the families (e.g. maternal year of birth, country of origin, partnership status, school education, vocational training, type of income) were documented. FMs also recorded the topics of their counseling activities (e.g. medical or nutritional counseling). At program entry and termination, the FMs furthermore documented the family's overall psycho-social characteristics using an initial and final checklist (pre- and post-intervention documentation). These checklists comprised seven problem areas (1. mother/parents [e.g. recognizable increased demand of support]; 2. biography [e.g. no understanding of German language]; 3. profession [e.g. missing school degree]; 4. family [e.g. missing social support]; 5. finances [e.g. severe financial constraints]; 6. parents/child [e.g. non-compliance with screening examinations]; 7. child [e.g. indications of child neglect]) with 3 to 5 subcategories each. The checklist was developed by the EP project leaders specifically for this FM service. However, a formal evaluation of this instrument was not performed.

The FMs rated the observed magnitude in each of these problem areas as either “none”, “low”, “moderate” or “high”. Based on these ratings, the local youth welfare office calculated a problem score for each area by assigning a value of 0 for "none" or "low", 1 for “moderate” and 2 for a “high” magnitude of the respective problem. This problem score formed the basis for the present analyses.

Due to different numbers of subcategories in each problem area, the scores had to be weighted. By summing up the weighted scores a total problem score was determined which ranged from 0 (minimum) to 54 (maximum). Finally, the difference between the initial and the final problem score values was categorized into a change variable (values < 0 = improvement; values of 0 = no change; values > 0 = worsening). A discernible impact was defined by a significant change of the total or subgroup problem scores.

### 2.3. Statistical analysis

Statistical analysis of quantitative data was performed for the entire sample as well as stratified by country of origin, partnership status and SES. The definition of SES based on the variables “school education”, “vocational training” and "income from work". Descriptive characteristics of the study sample are presented as frequencies or medians (25^th^ percentile; 75^th^ percentile). Tests for differences between exposure groups were performed using the Chi-Square-Test for categorical variables, and the Mann-Whitney-Test for continuous variables, including the problem scores assigned. Comparisons between the overall initial and final problem score were performed using the Wilcoxon-Test. A two-sided p-value of < 0.05 was considered statistically significant. Data were analyzed using the SPSS statistical software package version 21 (SPSS Inc., Chicago, IL, USA).

### 2.4. Qualitative data

For the qualitative, so-called expert interviews, all 16 FMs working in the model region were invited to participate. For an “expert interview”, the interviewed person is defined as an expert in its field of action, providing insights into her or his own actions or the investigated target group [78], [79]. Four FMs agreed to participate in the interviews.

All staff involved in obtaining and interpreting the qualitative data had no prior relations to the interviewees. Data acquisition and interpretation were carried out independently from the Youth Office staff. Scientific and Youth Office staffs were in no financial or otherwise tributary relation. The investigation was conducted strictly according to the Ethical Code of the German Sociological Association [80].

The interview guide was developed according to Helfferich [81]. A pre-test interview with one FM was performed to test the guideline. As no changes of the interview guide were necessary, this interview was included in the final analysis.

Overall, five main interview topics were addressed of which “insights into home visits”, “importance of diet and nutrition” and “perinatal programming” were further analyzed. The following key questions were used to address these topics: (1) “Please describe a typical working day as FM.” (2) “What is the relevance of diet and nutrition in your work as FM?” (3) “What is the relevance of perinatal programming in your consultation and support activities?”

The interviews were conducted by a female research assistant trained to perform these interviews, who was not in contact with the interviewees beforehand and had no personal relation to them. Before being interviewed, the FMs signed a consent form which allowed using the electronically recorded interview data in an anonymous form. The pre-test interview was not recorded electronically, and one FM refused the electronic recording of the interview. Therefore, for these two interviews only a handwritten interview protocol was available. All interviews were conducted between May and June 2013. Each interview lasted between 40 and 65 minutes. The electronically recorded interviews were verbatim transcribed according to Kowal et al. [82] using f4-software. To ensure comparability between interviews the interviewer followed the interview guideline and applied the same question style. After the interviews, data on age, professional experience and qualification using a standardized questionnaire were collected [83]. Other relevant information (e.g. interview atmosphere, conversation after turning off the audio recorder) was noted in a handwritten interview protocol [83].

The data analyses and interpretation was conducted mainly from a health science perspective. For the interview data analyses the qualitative content analysis approach by Mayring was chosen [84]. Therefore, a category system was developed deductively and a coding guideline was compiled. The interview data analyses were performed with MAXQDA-software. As a first analytical step, a test run was carried out to verify the category system, followed by a category system revision where necessary. Finally, the interview material was analyzed based on the finalized category system. The extracted relevant text passages were paraphrased, generalized and finally summarized according to Mayring [84]. The analyses were carried out by the first, third and senior author. The cited quotations were translated from German to English by the first author and cross-checked by the senior author.

## 3. Results

The study results are grouped according to the objectives mentioned above and are presented by comparing data from the quantitative and qualitative analyses [74].

Overall, quantitative data from 295 projects participants was available for this analysis. All participants who provided information were women. Almost a fifth (17%) of the participants had a migration background; most of them of Eastern European origin. Less than half of the participants (44%) lived in a partnership. Approximately two thirds had a low educational attainment (62%) and no complete vocational training (59%). Only 26% of the participants received income from work. These and further general characteristics of the study sample are presented in **Table 1**.

Four structured expert interviews with FMs (25% of all FMs in the model region) were conducted and analyzed. The interviewees had a median working experience of 29 years as midwife and three years as FM. General characteristics of the interviewees are presented in **Table 2**.

**Table 1.** Socio-demographic characteristics of the study sample, Early Prevention Project (n = 295).

Socio-demographic characteristics	
n (%) Women	295 (100.0)
Maternal age in years^a^	29 (25; 34)
n (%) Country of origin: Germany	242 (82.9)
n (%) Living in a partnership	130 (44.4)
n (%) Secondary and higher school education	108 (38.3)
n (%) Vocational training completed	110 (40.9)
n (%) Income from work	74 (26.1)

^a^ Data are median (25^th^; 75^th^ percentile). Missing values: n = 3 for country of origin; n = 2 for living in partnership; n = 13 for school education; n = 26 for vocational training; n = 11 for income from work.

**Table 2.** General characteristics of the interviewed family midwives, Early Prevention Project (n = 4).

Family midwives	
n (%) Women	4 (100.0)
n (%) Age	
41–50 years	1 (25.0)
51–60 years	3 (75.0)
Working experience as midwife (in years)^a^	29 (23; 35)
Working experience as family midwife (in years)^a^	3 (2; 6)

^a^ Data are medians (25^th^ percentile; 75^th^ percentile).

The above described characteristics of the families in the EP project are specified by information provided in the FMs interviews. It was mentioned that mothers participating in EP projects often reported financial problems (e.g. no income from work, liabilities), family disputes (e.g. violence, partnership trouble, problems relating to acknowledgment of paternity) or addiction problems (e.g. drug and/or alcohol addiction, relapse). In addition, the age of the mothers as well as migration status were mentioned as being critical.

*“They have quite different problems [than mothers not participating in EP projects].”* [A: Z 31]. *“In general, there are more existential problems.”* [A: Z 45]. *“Very often I look after […] very young mothers, […], that is 16 or 14 year old mothers.”* [A: Z 151–153]. *“In many cases the women are single mothers or change their partner quickly […].”* [C*: Z 100–101]. *“My focus is mainly in the care of women and families with migration background (e.g. African, Chinese women).”* [C*: Z 18–19]. *“[…] they have more financial problems.”* [C*: Z 31–32].

To determine the impact of the FMs activities (first objective), changes in the problem score were analyzed. Overall, the initial problem score (median [25^th^ percentile; 75^th^ percentile]) summed up to 16.5 [10.3; 24.0]. At the end of the FMs home visit phase it decreased to 7.3 [2.9; 17.4], resulting in a significant improvement of the total problem score of 6.7 points (*p* < 0.001). Based on this problem score, for 84% of the participants an improvement could be determined, while for 12% a worsening was observed and 5% remained unchanged (**Table 3**). These positive results are supported by comments of the FMs made during the interviews.

**Table 3.** Overall initial, final and change problem score values of the whole study sample, Early Prevention Project (n = 295).

Initial problemscore rating n = 292	Final problemscore rating n = 281	p^b^	Change problem score rating n = 278			
Median^a^	Median^a^		n (%) improvement	n (%) no change	n (%) worsening	Median^a^
16.5 (10.3; 24.0)	7.3 (2.9; 17.4)	<0.001	232 (83.5)	13 (4.7)	33 (11.9)	-6.7 (-11.2; -2.1)

^a^ Data are medians (25^th^ percentile; 75^th^ percentile). Missing values: n = 3 for initial problem score; n = 14 for final problem score; n = 17 for change problem score. ^b^Comparison between the overall initial and final problem score based on the Wilcoxon-Test for continuous variables.

*“Once, I supported a pregnant mother […], her apartment was completely filled with smoke […] and she was sitting right in the middle […]. Now, her child is three months old […] and they stopped smoking in the apartment […]”* [B: Z 517-523]. *“[After a nutritional counseling], I am very happy, when I see a couple of apples and strawberries in the kitchen. Something's happened!”* [B: Z 604-605]. *“I value such changes, even if they are small.”* [B: Z 516].

However, the FMs also mentioned that implementation of recommendations varied considerably. Recommendations were reported to be accepted and implemented only when they appeared to be connected to the reality of the family's life.

*“[The range is] from 150% implementation to […] complete ignorance.”* [B: Z 529-544]. *“The experiences are very different. There are mothers who fully implement the recommendations. For them I'm kind of a mom. And others think again "Let her prattle".”* [C*: Z 167-169]. *“Problems in the implementation [occur] […] if it [the change] doesn't make sense [for the families]. If it does not match their living environment.”* [B: Z 551-553].

**Figure 2.**
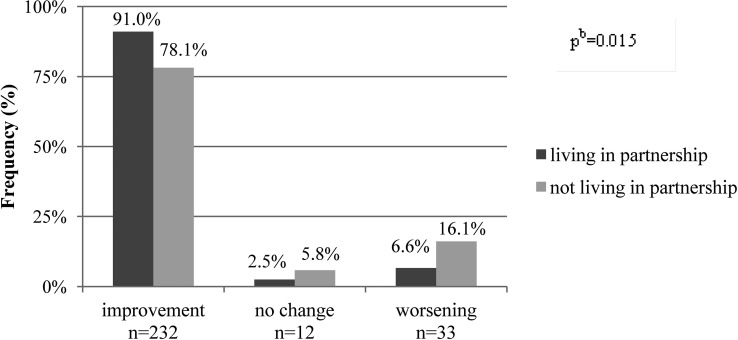
Categories of change in problem score values, stratified by partnership status, Early Prevention Project (n = 295)^a^ ^a^ Missing values: n = 17 for change problem score; n = 2 for living in a partnership. ^b^ Test for differences between partnership status for change problem score based on the Chi-Square-Test for categorical variables.

Regarding the second objective, to identify subgroups with specific and more intense intervention needs, the study sample of the quantitative analysis was stratified by country of origin as well as partnership, educational and income status. In **Table 4**, initial and final problem score values within these subgroups are displayed. Single parents (*p* = 0.004) as well as families with low SES (low school education, no vocational training and no income from work) (*p* < 0.001) had significantly higher problem score values at program entry compared to the respective comparison groups. Only for the variable “country of origin” no significant differences were observed. In all these subgroups, the problem scored increased significantly (*p* < 0.05; based on the Wilcoxon-Test) (data not presented). Although slight differences between strata observed at program entry remained (p < 0.05) also after the home visits, although slight differences in the magnitude of reported changes between subgroups were observed. Furthermore, single mothers seemed to benefit to a lesser extent compared to those living in a partnership (*p* = 0.015) (**Figure 2**). In contrast, neither migration status nor SES appeared to be related to differential problem score changes (data not presented). No respective information on this topic was found in the interviews.

**Table 4.** Initial and final problem score values stratified by country of origin, partnership and socio-economic status, Early Prevention Project (n = 295).

Subgroup	n total	Initial problem score rating n = 292		Final problem score rating n = 281		Change problem score rating n = 278
		Median^a^	p^b^	Median^a^	p^b^	Median^a^
Country of origin: Germany	242	16.5 (10.3; 24.0)	1.0	7.1 (2.9; 15.9)	0.2	-7.6 (-11.5; -2.5)
Country of origin: not Germany^c^	50	16.7 (10.8; 24.4)		10.0 (3.3; 21.9)		-4.6 (-9.1; -1.0)
Living in a partnership^d^	129	14.4 (9.7; 22.3)	0.004	5.4 (1.7; 14.2)	0.005	-6.8 (-10.8; -3.5)
Not living in a partnership^e^	162	19.2 (11.2; 26.4)		9.1 (4.2; 20.5)		-6.8 (-12.0; -0.7)
Secondary & higher school education^f^	106	12.8 (9.0; 18.1)	< 0.001	5.4 (1.4; 10.9)	< 0.001	-6.5 (-11.2; -2.9)
No secondary & higher school education^g^	174	20.4 (11.7; 28.1)		10.0 (4.5; 21.6)		-7.5 (-11.6; -2.0)
Vocational training completed	108	12.0 (8.5; 17.3)	< 0.001	4.2 (1.3; 8.8)	< 0.001	-7.0 (-10.8; -3.5)
Vocational training not completed	159	20.5 (12.7; 28.3)		11.2 (5.0; 21.9)		-6.8 (-11.8; -2.0)
Income from work	72	11.9 (8.0; 16.2)	< 0.001	4.6 (1.3; 7.6)	< 0.001	-6.8 (-10.3; -3.4)
No income from work^h^	210	19.4 (11.3; 27.2)		9.2 (4.2; 21.2)		-6.8 (-11.6; -2.0)

^a^ Data are medians (25^th^ percentile; 75^th^ percentile). Missing values: n = 3 for initial problem score; n = 14 for final problem score; n = 3 for country of origin; n = 2 for living in a partnership; n = 13 for school education; n = 26 for vocational training; n = 11 for income from work. ^b^ Test for differences between strata, within the sections of initial or final problem score, were performed using the Mann-Whitney-Test for continuous variables. ^c^ This comprises: people from Central and Northern Europe, the Middle East, Mediterranean countries, Eastern Europe, Africa, and other countries. ^d^ This comprises: married, not married couples. ^e^ This comprises: separated couples, divorced, single, widowed. ^f^ This comprises: college student, university entrance diploma, university degree. ^g^ This comprises: high school, special school, no degree. ^h^ This comprises: social welfare, pension, no income.

To determine the relevance of diet and nutrition-related topics within the current EP project (third objective), the provision of nutritional counseling, as documented by the FMs during their home visits, was analyzed. Nutrition counseling encompassed a wide range of topics such as maternal nutrition during and after pregnancy, breastfeeding, and preparation of formula. Nutritional counseling was demanded by 89% of the families. No significant differences in counseling demand consisted between strata (data not presented).

According to the reports of the FMs in the interviews, fundamental topics of their consultation were baby-care, advancement of child development and psycho-social support. As a further important focus of the consultations, health issues were mentioned, particularly diet and nutrition.

*“Well, in the area of health, of course, nutrition is an important component […].”* [A: Z 301f.]. *“Nutrition is of major importance […].”* [C*: Z 147]. *“My recommendations have the objectives to create good opportunities for the child's development […]. This includes also the diet of the child and mother […].”* [C*: Z 87–89].

The FMs reported that the supported families often brought up diet and nutrition-related topics themselves. In terms of diet and nutrition-related activities during the consultations, FMs mentioned the assessment of maternal dietary and purchasing behavior. They reported to give advice on optimized food selection during or after pregnancy.

*“Questions that are asked […] especially during pregnancy include: What may I eat and what not?”* [A: Z 422–423]. *“In principle diet is always an issue. Questions dealt with were "What causes winds?" or "What is accepted by the child?” I also explain what should be eaten during pregnancy.”* [C*: Z 144–146]. *“[...] I ask them how, what do you eat; tell me, for breakfast, lunch and dinner. What do you eat in-between and what do you drink?”* [B: Z 639–641]. *“Some mothers just eat at lunch for the first time a day. So all I care about is that they eat regularly. [...] I'm trying to achieve that mothers eat more fresh stuff, not just fast food.”* [A: Z 273–278].

Advices regarding the initiation of breastfeeding, breastfeeding problems and infant nutrition were also reported. However, due to the fact that most of the home visits started in the postnatal period and thus after formula feeding initiation, FMs reported that recommendations concerning hygienic preparation and correct dosage of infant formula were of much higher importance.

*“So, when I'm already in the family during pregnancy, then it is about […] breastfeeding yes or no […].”* [A: Z 225–228]. *“Before we come into the families, most mothers had already weaned.”* [D*: Z 44]. *“But also hygiene is an issue as well, especially in the family midwifery. How am I actually preparing a bottle? How, […] clean should it be?”* [A: Z 233–235].

Although the FMs considered the pre- and postnatal phase to be an appropriate time period for introducing lifestyle modifications, no respective nutritional content was taught in the FM qualification seminars. The FMs indicated that they acquired some nutritional expertise from reviewing literature.

*“I assume and experience that all women want to have a healthy […] child. […] they all want the very best for their child. So I have a good chance to get involved and to motivate through little things.”* [B: Z 597–602]. *“I think by the fact that you've built mutual trust they [the families] would do this [healthy nutrition] rather than when they [the families] are given a booklet […]. […] [This is a] larger effect that the family midwives have in the family to influence the eating habits.”* [A: Z 466–470].

The FMs emphasized that alteration in dietary behavior is challenging and time intensive. According to their experience, counselling and interventions should be adapted to the knowledge and skills of the individual families. Due to the necessary (but only limited own expertise) introduction of nutrition experts for further counselling is regarded as an option. Since home visits through midwives were not called into question by the families, additional support provided by a nutrition expert might by accepted more easily.

*“This I really have to say, this is often quite difficult. For various reasons: First of all, because they're not used to it [fruits, whole grain products]. Secondly, there is not enough money or it is not taken for it.”* [A: Z 284–286]. *“She has neither cooked nor does she know how to cook. […] this mother had an additional assistance […] who taught her how to cook.”* [A: Z 313–317]. *“I also give some initial advice or I refer the mother to a “food expert”. I also delegate such topics.”* [B: Z 684–686]. *“Recommendations for lifestyle changes should only be given in small steps […] in order not to overburden the families. So I try not to switch […] the complete diet immediately.”* [C*: Z 123–133]. *“Mothers must be addressed according to their current knowledge.”* [C*: Z 198–199]. *“One of my main functions is to connect the families to other counselling centers which can further help them.”* [C*: Z 163–164].

*“We are midwives with a wider perspective. That means I go to the families […] and they know the midwife comes.”* [B: Z 15–16]. *“The personal relationship is the ‘most important thing’”*. [C*: Z 195–196]. *“Trust establishment is very important.”* [D*: Z 25]. *“If I tell a mother, let us take the child's clothes off and weigh it […], then that is a normal midwife's work. And if I notice a lack of hygiene or a bruise […], I can observe it along the way. But if the youth office tells a mother to take the child's clothes off […] she is spoiled for a fight.”* [B: Z 199–201].

The interviews further revealed that FMs were not familiar with the term perinatal programming. However, some potential adverse programming effects (e.g. gestational diabetes) were mentioned.

*“Upon the occurrence of diabetes during pregnancy […], I advise the mothers to eat less sweet thinks and to drink less sweet drinks, […] because most do not know this. In addition I send the women to diabetes counseling.”* [C*: Z 157–160]. *“[…] children from mothers who have undetected gestational diabetes also have an increased risk for diabetes. […] and this can then be reduced by breastfeeding.”* [A: Z 515–519].

## 4. Discussion

This study set out to determine whether the existing access to a difficult-to-reach population group, that are vulnerable and disadvantaged families via FMs offers an opportunity to introduce health and nutrition related preventive activities. According to the performed quantitative and qualitative analyses, it is concluded that this access is a promising entry point for further intervention services. This conclusion is based on the results of the analyses addressing the three posed research questions. According to the program documentation data collected by the FMs, a positive impact on the family's development could be described during their consulting service. While overall an improvement among the participating families was seen, single mothers appeared to profit less and might therefore be a subgroup in need of more specific interventions. Finally, diet and nutrition-related topics were regarded to be important by both the families and the FMs. While the FMs established an easy and trustful access to the families, an extensive support and network with nutrition experts seem to be required.

Regarding the impact of the FMs service, similar conclusions have been drawn from both national [69]–[71] and international studies of “home visiting services” [61]–[65], [67], [68]. Overall, these studies stated that home visits had a positive impact on the social development of the child and on infant care. However, only few studies attempted to quantitatively describe observed changes in problem characteristics. To our best knowledge, only one study has also shown an improvement by using a problem score [71]. Further, potential subgroups with specific assistance requirements within these projects are rarely analyzed. This analysis indicated that within the target group subgroups might exist which appear to benefit less from the FMs service. Particularly single mothers may represent a target group with higher and probably more specific service demands. Single parenting has previously been described as a risk factor for financial constraints and suboptimal health behavior [85], [86]. Conversely, a stable partnership has been described as supportive for a favorable child and family development [87]. Thus, FMs home visits might have to be more specifically tailored to this subgroup of single mothers. Several other studies also reported positive effects of social support (e.g. through home visitation) [85], [88], [89]. For example, a RCT indicated that mothers who took part in a program of “home visiting services” were more likely to live in a stable partnership in the future [67], [90].

With respect to the relevance of diet and nutrition-related topics among families and FMs, FMs reported that for them and, in their opinion, also for the supported families, these were important topics during pregnancy and early childhood. This was documented by the high frequency of demanded nutrition counseling by the families. The relevance of diet and nutrition to midwives and FMs was also documented by other studies [91], [92]. Since maternal diet and nutrition during pregnancy, especially among mothers with low SES, do often not meet current recommendations [34], [35], this aspect is of major public health importance when adverse effects of perinatal programming are to be reduced. Hence, the observed interest in diet and nutrition in the target group is a crucial prerequisite for future implementation of effective diet and nutrition-related interventions. Similarly, perceiving diet and nutrition-related topics to be important is a fundamental prerequisite for involving FMs into respective additional intervention activities. However, despite the noticed interest, the implementation of dietary recommendations seemed to be difficult for the FMs. Therefore the strengthening of the cooperation and collaboration between FMs and nutritional experts could be an important component to implement health and nutrition related activities.

On the one hand, the collaboration could be beneficial regarding the FM's nutritional expertise. The need for nutritional training has already been described before [92]. On the other hand, a tighter network would help to recommend appropriate nutrition experts to families. Therefore, a closer collaboration with nutrition experts could further increase the quality of the home visits.

Given the fact that in this EP project FMs started their home visits mostly after birth of the child, breastfeeding initiation and maintenance were less relevant during consultations. Instead, FMs focused on the hygienic preparation and correct dosage of infant formula. This was also observed in another study [92]. Since the interviewed FMs reported that they perceived women in the pre- and postnatal phase as very accessible to behavioral change towards health promotion, efforts should be made to start home visits earlier. In this context, also other studies described that receiving home visit services during pregnancy increased breastfeeding initiation [68], [93]. Numerous studies already documented the beneficial effects of breastfeeding and its positive effects on both the child's and the mother's metabolic development [40], [41], [94]–[100].

Finally, our results also point out that a trustful and respectful relationship appears to be an important prerequisite for working with vulnerable and disadvantaged families. According to the FMs perception, their very personal and easy access is the most important reason for their acceptance as well as for the acceptance of recommendations. This impression is supported by studies dealing with reasons for non-attendance or premature termination of participation of families in similar projects and interventions. A lack of trust and/or misperceived project intentions have been identified as major reasons of project failure [56], [57]. Therefore, attention should be put on appreciation and respect of the families for building a trustful relationship. Hence, FMs could act as a gateway for a trustful access to families, which then would allow implementing health-related interventions.

Altogether, the suggested activities might help to reduce health inequality and adverse perinatal programming in vulnerable and disadvantaged families. The underlying hypothesis of this study assumed that the frequently observed transgenerational transmission of health and social inequality is, beyond societal and political factors, also attributable to the biological effects of perinatal programming. This hypothesis has been confirmed in the context of severe malnutrition as it is observed in developing countries [101], [102]. Therefore, preventive and health-promoting interventions during the time window between conception and approximately the second year of life are considered most effective [103]. Given the increasing incidence of type 2 diabetes mellitus, obesity, and the rising prevalence of high-risk lifestyle behavior (e.g. smoking, unfavorable diet, physical inactivity) [9], [10], [12]–[18], [104], [105] as well as lower use of preventive and health-promoting services of socially disadvantaged people [106]–[108], the reduction of adverse perinatal programming effects represents an important public health target. So far, the long-term effects of “home visiting services” are only rarely examined. The few studies available indicate promising effects. A community health program existing since 1977, offering prenatal and infancy home visits for low-income, first-time mothers and their children prospectively assessed life-style and health outcomes. 12 years after the intervention a healthier lifestyle of the children and fewer internalizing disorders compared to a control group were observed [109]. In addition, the maternal life situation improved and governmental spending for the family fell significantly [67]. These studies underline the potential of early life support and interventions.

Our study has several limitations. The estimation of problem score items was based only on the subjective judgment of the FMs delivering the service; neither an external nor the program recipient perspective could be taken into account. Since their final problem score assessment also implied an appraisal of their own work, an over-reporting of positive effects has to be assumed. However, the analysis of the problem score items was a secondary analysis of existing data, implying that no influence on content and scaling of the assessed data by the FMs was possible. Further limitations are that none of the documentation instruments used in the EP projects had been tested for validity and reliability. In addition, since the problem score items included measures of social and family status, stratified analyses have to be interpreted with caution. For example, the fact that single mothers appeared to improve to a lesser extent might be due to the circumstances that single parenthood resulted in lower problem score values. Since a change in family status is beyond the influence of the FMs, a lower improvement in score values was to be expected. Another critical aspect is the “deficit-based approach” underlying the checklist which took strengths and resilience factors of the families not into account. Therefore, the problem score should only be regarded as a rough indicator of FMs service impact and should not be interpreted as an evaluation of effectiveness or true effects sizes. Moreover, no further health parameters, such as gestational age, birth weight and breast feeding initiation were available. Furthermore, the qualitative analysis is based on four interviews only. More interviews might be needed to enlarge and confirm the current conclusions or to identify new aspects.

The strength of the current study lies in the rare data source as well as its size, allowing stratification of the sample, which gave hints towards differential consultation effects and options for better intervention tailoring. Information from low income and marginalized population groups is rare and difficult to obtain [110]–[113].

## 5. Conclusion

EP projects offer an easy and trustful access to vulnerable and disadvantaged families and could therefore be an effective option to reach these population subgroups with further health and nutrition intervention services. Thereby, a reduction of health inequality via reduced adverse perinatal programming might be achieved.

An earlier start of home visits, preferably already during pregnancy, would allow putting additional focus breastfeeding promotion. Addressing specific needs of single parents could strengthen these efforts.

Implementing intensified health and nutrition counselling in this setting investigated here would require a tighter interdisciplinary collaboration with health and nutrition experts. Furthermore, the sustainability of EP projects, as well as the identification of future periods in which continued support might be needed, should be evaluated. Long-term monitoring and continuing evaluation are therefore to be established.
